# Outcomes in a Cohort of Women Who Discontinued Maternal Triple-Antiretroviral Regimens Initially Used to Prevent Mother-to-Child Transmission during Pregnancy and Breastfeeding—Kenya, 2003–2009

**DOI:** 10.1371/journal.pone.0093556

**Published:** 2014-04-14

**Authors:** Timothy D. Minniear, Sonali Girde, Frank Angira, Lisa A. Mills, Clement Zeh, Philip J. Peters, Rose Masaba, Richard Lando, Timothy K. Thomas, Allan W. Taylor, for the Kisumu Breastfeeding Study Team

**Affiliations:** 1 Epidemic Intelligence Service, CDC, Atlanta, Georgia, United States of America; 2 Division of HIV/AIDS Prevention, CDC, Atlanta, Georgia, United States of America; 3 Department of Infectious Diseases, St. Jude Children's Research Hospital, Memphis, Tennessee, United States of America; 4 CDC Information Management Services, ICF International, Atlanta, Georgia, United States of America; 5 Kenya Medical Research Institute/CDC Research and Public Health Collaboration, Kisumu, Kenya; University of Southampton, United Kingdom

## Abstract

**Background:**

In 2012, the World Health Organization (WHO) amended their 2010 guidelines for women receiving limited duration, triple-antiretroviral drug regimens during pregnancy and breastfeeding for prevention of mother-to-child transmission of HIV (tARV-PMTCT) (Option B) to include the option to continue lifelong combination antiretroviral therapy (cART) (Option B+). We evaluated clinical and CD4 outcomes in women who had received antiretrovirals for prevention of mother-to-child transmission and then discontinued antiretrovirals 6-months postpartum.

**Methods and Findings:**

The Kisumu Breastfeeding Study, 2003–2009, was a prospective, non-randomized, open-label clinical trial of tARV-PMTCT in ARV-naïve, Kenyan women. Women received tARV-PMTCT from 34 weeks' gestation until 6-months postpartum when women were instructed to discontinue breastfeeding. Women with CD4 count (CD4) <250cells/mm3 or WHO stage III/IV prior to 6-months postpartum continued cART indefinitely. We estimated the change in CD4 after discontinuing tARV-PMTCT and the adjusted relative risk [aRR] for factors associated with declines in maternal CD4. We compared maternal and infant outcomes following weaning–when tARV-PMTCT discontinued–by maternal ARV status through 24-months postpartum. Compared with women who continued cART, discontinuing antiretrovirals was associated with infant HIV transmission and death (10.1% vs. 2.4%; *P* = 0.03). Among women who discontinued antiretrovirals, CD4<500 cells/mm3 at either initiation (21.8% vs. 1.5%; P = 0.002; aRR: 9.8; 95%-confidence interval [CI]: 2.4–40.6) or discontinuation (36.9% vs. 8.3%; P<0.0001; aRR: 4.4; 95%-CI: 1.9–5.0) were each associated with increased risk of women requiring cART for their own health within 6 months after discontinuing.

**Conclusions:**

Considering the serious health risks to the woman's infant and the brief reprieve from cART gained by stopping, every country should evaluate the need for and feasibility to implement WHO Option B+ for PMTCT. Evaluating CD4 at antiretroviral initiation or 6-months postpartum can identify pregnant women who would most benefit from continuing cART in settings unable to implement WHO Option B+.

## Introduction

In 2012, the World Health Organization (WHO) amended their 2010 guidelines for women receiving triple-antiretroviral drugs for prevention of mother-to-child transmission of HIV (tARV-PMTCT) (Option B) to include the option to continue combination antiretroviral therapy (cART) indefinitely (Option B+) [1]. This option was formally included in the WHO 2013 consolidated guidelines for the management of HIV. Operationalizing Option B+ for all pregnant women may be challenging in resource constrained settings. The Kesho Bora study, which compared Option B (zidovudine, lamivudine, and lopinavir/ritonavir) with zidovudine until delivery with single-dose, peripartum nevirapine, demonstrated that women's CD4 count returned to baseline approximately six months after discontinuing triple-antiretroviral therapy [2]. However, data directly comparing the maternal benefits of Option B+ with Option B have not been published. We evaluated maternal clinical, infant, and immunological outcomes over 24 months among women participating in a clinical trial stratified by whether women discontinued antiretrovirals at 6 months postpartum or continued cART for their own health.

## Methods

### Human Subjects Approval

The study was approved by the ethical review committees of the Kenya Medical Research Institute (KEMRI) and the US Centers for Disease Control and Prevention (CDC).

### Design

The Kisumu Breastfeeding Study (KiBS), 2003–2009, evaluated tARV-PMTCT; women received zidovudine, lamivudine and either nevirapine or nelfinavir from 34 weeks' gestation to 6 months postpartum when they discontinued tARV-PMTCT and breastfeeding (Clinical Trials registration number NCT00146380) [3]. Women with CD4 T-lymphocyte count (CD4) ≤250 cells/mm^3^ at 6-months postpartum and women with either CD4<200 cells/mm^3^, any WHO Stage IV diagnosis, or CD4<350 cells/mm^3^ with any WHO Stage III diagnosis at initiation or any point prior to 6 months postpartum all continued cART indefinitely for their own health. Women who met the above criteria after 6 months postpartum were eligible to restart cART indefinitely for their own health. Mothers and infants were followed until 24 months postpartum. We evaluated the prevalence of maternal tuberculosis, maternal death, infant HIV infection, infant death, and loss-to-follow-up from 6 months until 24 months postpartum. Infant HIV status was ascertained by HIV DNA PCR [3]. We also determined risk for steep decline of maternal CD4 following cessation of tARV-PMTCT based on CD4 at initiation, CD4 at discontinuation, and viral load (VL) suppression (<200 copies/mL) at discontinuation.

### Statistical Methods

Analysis was performed using SAS 9.2 (SAS Institute, Inc., Cary, NC). We used Fisher's exact test to compare categorical outcomes. We used a mixed linear model (PROC GENMOD) to estimate the change in maternal CD4 from 6 months to 24 months postpartum for three groups defined a priori: women who had initiated tARV- PMTCT with CD4<500 cells/mm^3^ and stopped with CD4≥500 cells/mm^3^ (A: <500/≥500; n = 165), women who had both initiated and discontinued tARV-PMTCT with CD4>500 cells/mm^3^ (B: ≥500/≥500; n = 131), women who had both initiated tARV-PMTCT with CD4<500 cells/mm^3^ and discontinued with CD4 between 350–500 cells/mm^3^ (C: <500/350–500; n = 49). We right-censored women who restarted tARV for any reason at the date they restarted. Among women with CD4>350 cells/mm^3^ at antiretroviral discontinuation, we used a log-binomial model to estimate the adjusted relative risk of CD4 count decreasing to <350 cells/mm^3^ within 6 months of stopping tARV-PMTCT stratified by CD4 at initiation of tARV, CD4 at cessation of tARV, and VL suppression at cessation of tARV.

## Results

Of the 522 women who enrolled in KiBS, 22 did not deliver [2], 28 withdrew consent, 15 were not included in this analysis due to death of mother (2) or infant (13) prior to 6 months postpartum, 9 were lost-to-follow-up, and 448 women-infants pairs were still enrolled at 6 months postpartum: 82 women continued cART and 366 women discontinued tARV-PMTCT; 21 (6%) of these were right censored for re-initiating cART between 6 and 24 months postpartum. The median maternal age at enrollment was 26 years (interquartile range [IQR]: 22–30) among women who continued cART and 23 years (IQR: 20–27) among women who stopped tARV-PMTCT (p = 0.0002). Fewer women who discontinued tARV-PMTCT were on nevirapine-containing regimens, as expected due to the precaution against the use of nevirapine in women with CD4 count >250 cells/mm^3^. Table 1 displays the baseline characteristics of the 448 women. Table 2 displays the adverse outcomes after 6 months postpartum by tARV status. Discontinuing tARV was significantly associated with an increased risk of infant death (risk difference: +5.5%; 95%-confidence interval [CI]: +1.1% to +9.8%) or HIV infection (Table 2) but not with maternal clinical outcomes. The association with infant death or HIV infection remained after adjusting for maternal CD4 at initiation and 6 months postpartum (P = 0.03). The median time to adverse event after discontinuing tARV was 83 days (interquartile range: 34–173 days).

**Table 1:** Characteristics of the 448 women included in the analysis of post-weaning outcomes — Kisumu, Kenya, 2003–2009.

	tARV-PMTCT Prophylaxis Regimen Continued as cART (N = 82)	tARV-PMTCT Prophylaxis Discontinued (N = 366)	*p*-value
Maternal Age, years (median, IQR)	26 (22–30)	23 (20–27)	0.0002
Parity (median, IQR)	1 (1–1)	1 (1–1)	0.08
Nevirapine Regimen, (n, %)	77 (94%)	221 (60%)	<0.0001
CD4 count, cells/mm3 (median, IQR)	161 (116–200)	444 (337–601)	<0.0001

**Table 2:** HIV-associated adverse events occurring after 6 months postpartum among women enrolled in the Kisumu Breastfeeding Study — Kisumu, Kenya, 2003–2009.

	tARV-PMTCT Prophylaxis Regimen Continued as cART (N = 82)	tARV-PMTCT Prophylaxis Discontinued (N = 366)	*p*-value (Exact)
***Clinical Outcomes***			
Maternal Death	2.4% (2)	1.6% (6)	0.89
Maternal Diagnosis of Tuberculosis	2.4% (2)	2.2% (8)	0.89
Loss-To-Follow-Up	0.0% (0)	2.2% (8)	0.20
***Infant Outcomes***			
Infant Death	2.4% (2)	7.9% (29)	0.09
Infant HIV Infection	0.0% (0)	2.5% (9)	0.38
Infant Death or HIV Infection	2.4% (2)	10.1% (37)^A^	0.03

A 1 infant was both HIV-infected and later died; only counted as 1 event.

The two women who died while continuing tARV as cART after weaning never achieved a CD4 count >500 cells/mm^3^ (maximum 453, 424). The cause of death was stated as AIDS dementia complex and meningitis. Of the six women who died after discontinuing tARV-PMTCT prophylaxis, two died due to acute dehydration (gastroenteritis), two died due to cardiopulmonary failure (rheumatic heart disease, congenital heart disease), and the cause of death was unknown for two. One woman died before her infant died three months later, and one infant died before his mother died seven months later.

**Figure 1:**
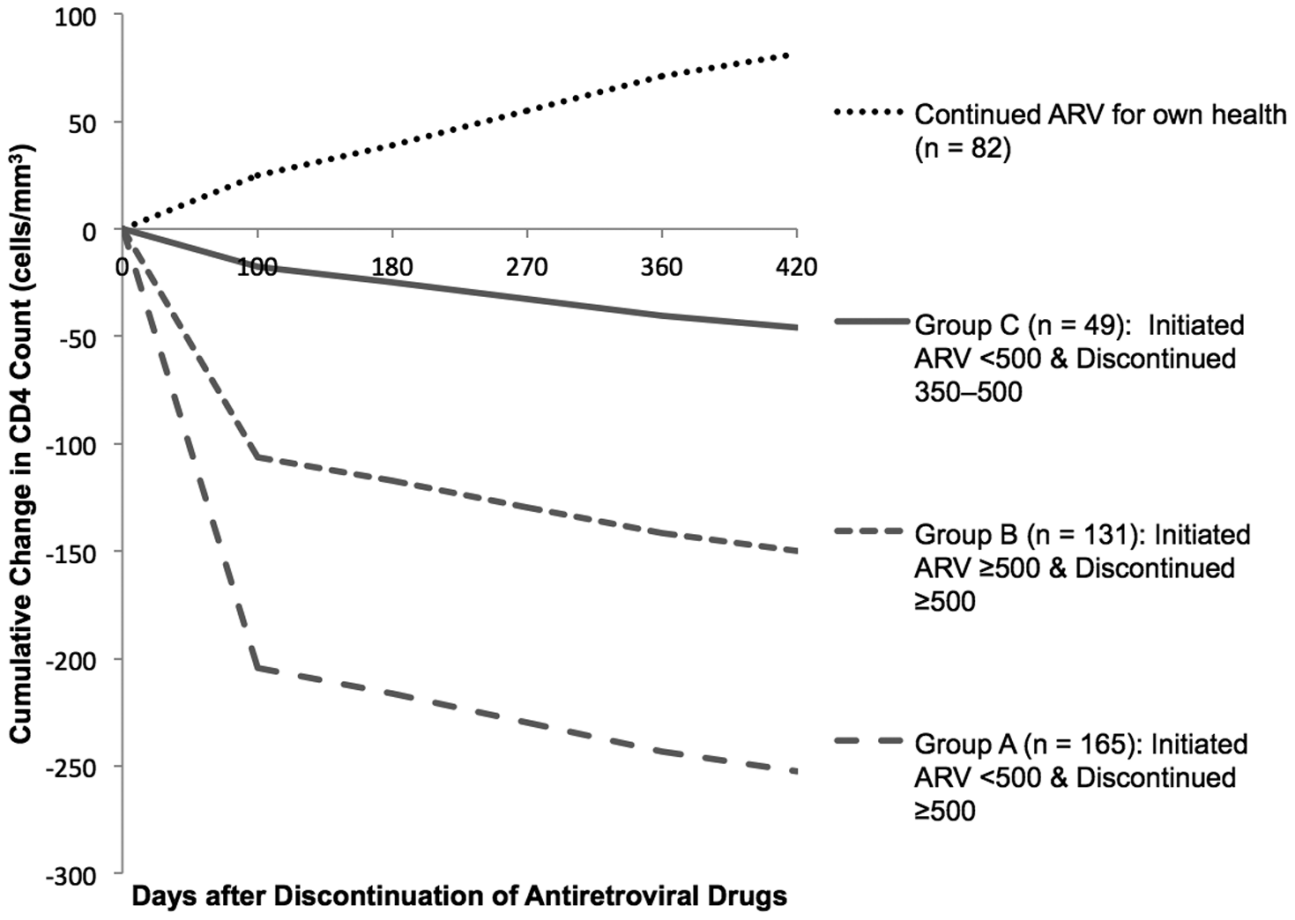
Rate of change in CD4 count over time by CD4 at initiation and discontinuation. Slopes of decline for each segment (≤3 months/>3 months) are non-parallel (P<0.05) (PROC MIXED). Group A (<500/≥500) median CD4 was 383 (range: 216–492) at initiation and 702 (range: 501–1432 at discontinuation. Group B (≥500/≥500) median CD4 was 631 (range: 500–1165) at initiation and 912 (range: 510–1867) at discontinuation. **Group C (<500/<500) median CD4 was 328 (range: 120–482) at initiation and 437 (range: 363–497) at discontinuation.**

During the first 3 months after discontinuing antiretrovirals, women in Group A (<500/≥500) demonstrated the fastest drop in CD4 after stopping tARV-PMTCT (−61 cells/mm^3^/month; Figure 1). Women in Group B (≥500/≥500) experienced a drop in CD4 after stopping antiretrovirals of −32 cells/mm^3^/month (Figure 1). Women in Group C (<500/350–500) experienced the slowest drop (−5 cells/mm^3^/month; Figure 1). Between 3 months and 18 months after discontinuing antiretrovirals, the CD4 decline for all three groups plateaued (Group A & B: −4 cells/mm^3^/month; Group C: −3 cells/mm^3^/month; Figure 1). Twelve women had initiated tARV-PMTCT with CD4>500 cells/mm^3^ and stopped with CD4 between 350–500 cells/mm^3^ (data not shown). Rise in log VL after discontinuing tARV was not different between the groups (Group A: 0.04 log copies/mL/month; Group B: 0.02 log copies/mL/month; Group C: 0.05 log copies/mL/month; P>0.05).

Women who initiated at CD4<500 cells/mm^3^ were far more likely to experience a drop to <350 cells/mm^3^ within 6 months of stopping tARV-PMTCT, independent of CD4 when tARV-PMTCT was discontinued, compared to women who initiated at CD4≥500 cells/mm^3^ (21.8% vs. 1.5%; aRR: 9.8; 95%-CI: 2.4–40.6). Women who stopped tARV-PMTCT with CD4 between 350–500 cells/mm^3^ were also more likely to experience a drop to <350 cells/mm^3^ within 6 months, independent of CD4 at tARV-PMTCT initiation, compared to women who stopped at CD4≥500 cells/mm^3^ (36.9% vs. 8.3%; aRR: 4.4; 95%-CI: 1.9–5.0). Women with a non-suppressed VL at discontinuation of tARV-PMTCT were more likely to drop to <350 cells/mm^3^ within 6 months than women with suppressed VL (21.2% vs. 11.4%; aRR: 1.6; 95%-CI: 1.0–2.6).

At enrollment, 82/448 (18%) women qualified to continue cART. Under the 2010 WHO antiretroviral treatment criteria [3], 127/448 (28%) additional women qualified to start cART within 18 months of stopping tARV-PMTCT, most (65%) within the first 6 months. At initiation of tARV-PMTCT, 216/448 (48%) women who did not yet qualify for continued cART had CD4<500 at initiation and 8 (2%) more had CD4<500 at discontinuation.

## Discussion

Like the Kesho Bora Study, women who discontinued triple-antiretroviral therapy had a rapid decline in CD4 [2]. Our findings demonstrate that decline is greatest in the first 3 months after discontinuing and that both initial and weaning CD4 influence the rate and depth of decline. Women who initiated tARV-PMTCT with CD4<500 cells/mm^3^ and discontinued with CD4 between 350–500 cells/mm^3^ (Group C) were at the greatest risk of meeting current WHO treatment criteria [4] within 6 months of stopping antiretrovirals. However, the women who initiated tARV-PMTCT with CD4<500 cells/mm^3^ and discontinued with a CD4≥500 cells/mm^3^ (Group A) were still at significant risk of meeting WHO treatment criteria within 6 months of stopping antiretrovirals compared to women who initiated tARV-PMTCT with CD4≥500 cells/mm^3^ (Group B). This sharp decline in CD4 upon stopping antiretrovirals may reflect a rebound towards a low pre-cART set point, which 7–8 months of tARV-PMTCT were insufficient to affect, and suggests that women with CD4<500 cells/mm^3^ either at tARV-PMTCT initiation or at 6 months post-partum should continue cART for their own health.

Furthermore, even though the mothers who continued cART did so because of advanced HIV infection (CD4<200 cells/mm3 or WHO stage III/IV disease), it was infants of mothers who discontinued antiretrovirals who were more likely to become HIV infected or die. While infant HIV infection as an independent outcome was not statistically significant by chi-square analysis, it is notable that all of the postpartum infant HIV infections occurred only among the women who discontinued tARV (Table 2). The infant HIV infections that occurred may be due to continued, unreported breastfeeding, a phenomenon which has been reported elsewhere [5]. If this is the case, lower breast milk VL could explain the reduction in infant HIV-infection among the women who continue cART [3], [6]. In addition, the increase in infant deaths was clinically important (+5.5%; 95%-CI: +1.1% to +9.8%), although not statistically significant (Table 2). The most common causes of infant death in our cohort were diarrhea (35%), pneumonia (16%), and respiratory failure (12%) [3]. Increased mortality has been observed among HIV-uninfected children <10 years old living in households with HIV-infected adults who received home-based cotrimoxazole prophylaxis alone compared with households where the adults received home-based cART and prophylaxis [7]. Improved immune defense among the women continuing antiretroviral therapy may have reduced maternal infections that may have otherwise been transmitted to the infant, particularly diarrheal disease and respiratory viral infections. The survival benefit among infants of mothers continuing antiretroviral therapy may also be due to improved milk production and consequently better nutrition, greater ability to care for the infant/child, and ability to contribute to household and income [7]–[8].

Early initiation of cART benefits the mothers, their infants and the population [7]–[10]. Despite the limitation of a relatively small sample size compared with the frequency of events, discontinuing antiretrovirals, in our study, was significantly associated with risk of infant HIV infection and death within the first 6 months after the mother stopped antiretrovirals. Furthermore, the women who initiated or stopped with CD4<500 cells/mm^3^ were at significantly increased risk of meeting WHO treatment criteria within 6 months after discontinuing tARV-PMTCT. Evaluating the CD4 at both initiation and 6 months postpartum can identify women who would benefit the most from continuing cART for their own health in settings unable to universally implement WHO Option B+. Using the thresholds in this analysis as an example, the number of women continuing cART after 6-months postpartum would increase from 82 (18%) to 224 (50%), costing an additional $9088 (first-line regimen cost: $64/year [11]). However, 106 (47%) of these additional women would have required cART for their own health within 12 months. Considering the serious health risks to the woman's infant, the brevity of the reprieve from cART gained by stopping, and predicted cost-effectiveness of WHO Option B+ [11]–[13], every country should evaluate the need for and feasibility to implement WHO Option B+ for PMTCT.

### Disclaimer

The findings and conclusions in this article are those of the authors and do not necessarily represent the views of the CDC. Use of trade names is for identification purposes only and does not constitute endorsement by the CDC or the Department of Health and Human Services.
